# Supplementary material for: MEME-LaB: motif analysis in clusters
Source: Bioinformatics. 2013 May 14;29(13):1696–7. doi: 10.1093/bioinformatics/btt248 (PMC3694638; doi:10.1093/bioinformatics/btt248)
Supplement: Supplementary Data [file supp_btt248_Supplementary_Data.zip › Supplementary_Data/Results_Files/which_gene_ids/cluster38_motif1.html]

Cluster 38 Motif 1


Cluster 38 Motif 1 is found in the following promoters

| Gene ID | Strand | Position\* |
| --- | --- | --- |

|  |  |  |
| --- | --- | --- |
| AT1G02000 | + | 434/500 |
| AT1G03390 | - | 439/500 |
| AT1G03610 | + | 364/500 |
| AT1G04000 | - | 114/500 |
| AT1G04960 | + | 102/500 |
| AT1G04970 | + | 19/500 |
| AT1G05270 | + | 293/500 |
| AT1G05940 | + | 286/500 |
| AT1G07350 | - | 120/500 |
| AT1G07750 | + | 91/500 |
| AT1G09140 | + | 37/500 |
| AT1G09180 | - | 442/500 |
| AT1G09960 | - | 95/500 |
| AT1G10500 | + | 137/500 |
| AT1G12200 | + | 8/500 |
| AT1G12310 | - | 286/500 |
| AT1G12810 | + | 199/500 |
| AT1G12820 | + | 225/500 |
| AT1G13400 | + | 250/500 |
| AT1G14530 | + | 487/500 |
| AT1G15170 | + | 119/500 |
| AT1G15600 | - | 113/500 |
| AT1G16470 | - | 481/500 |
| AT1G16700 | - | 160/500 |
| AT1G18410 | - | 203/500 |
| AT1G18720 | - | 481/500 |
| AT1G20090 | + | 439/500 |
| AT1G20880 | - | 245/500 |
| AT1G21370 | + | 70/500 |
| AT1G21840 | + | 320/500 |
| AT1G22640 | + | 288/500 |
| AT1G22790 | - | 208/500 |
| AT1G22985 | - | 160/500 |
| AT1G23100 | + | 151/500 |
| AT1G24180 | + | 55/500 |
| AT1G27000 | + | 251/500 |
| AT1G27100 | - | 476/500 |
| AT1G27170 | + | 366/500 |
| AT1G29160 | + | 161/500 |
| AT1G29195 | + | 482/500 |
| AT1G29800 | + | 400/500 |
| AT1G30130 | - | 43/500 |
| AT1G30930 | - | 142/500 |
| AT1G31350 | - | 5/500 |
| AT1G32710 | - | 26/500 |
| AT1G32950 | - | 485/500 |
| AT1G33250 | + | 205/500 |
| AT1G43890 | - | 194/500 |
| AT1G48470 | - | 221/500 |
| AT1G50370 | + | 260/500 |
| AT1G50940 | + | 75/500 |
| AT1G51350 | + | 49/500 |
| AT1G52550 | - | 312/500 |
| AT1G52680 | + | 267/500 |
| AT1G54290 | + | 137/500 |
| AT1G54530 | + | 317/500 |
| AT1G56450 | + | 137/500 |
| AT1G60430 | + | 387/500 |
| AT1G62040 | + | 320/500 |
| AT1G62790 | + | 420/500 |
| AT1G63090 | + | 104/500 |
| AT1G63450 | - | 94/500 |
| AT1G64040 | + | 198/500 |
| AT1G64740 | + | 353/500 |
| AT1G65000 | + | 320/500 |
| AT1G67160 | - | 298/500 |
| AT1G67195 | + | 127/500 |
| AT1G68680 | - | 61/500 |
| AT1G69410 | + | 412/500 |
| AT1G69450 | + | 204/500 |
| AT1G71180 | + | 437/500 |
| AT1G71950 | - | 272/500 |
| AT1G71960 | + | 252/500 |
| AT1G73920 | - | 30/500 |
| AT1G75270 | + | 383/500 |
| AT1G76770 | + | 310/500 |
| AT1G77610 | + | 57/500 |
| AT1G77770 | + | 458/500 |
| AT1G77890 | + | 479/500 |
| AT1G78955 | + | 151/500 |
| AT1G79160 | + | 244/500 |
| AT1G79210 | - | 487/500 |
| AT1G80040 | + | 371/500 |
| AT1G80230 | - | 239/500 |
| AT2G01490 | - | 77/500 |
| AT2G01850 | + | 173/500 |
| AT2G02380 | + | 489/500 |
| AT2G03110 | - | 263/500 |
| AT2G03980 | - | 31/500 |
| AT2G04020 | - | 358/500 |
| AT2G07640 | - | 261/500 |
| AT2G07671 | + | 261/500 |
| AT2G12290 | + | 483/500 |
| AT2G14170 | - | 463/500 |
| AT2G15310 | - | 479/500 |
| AT2G16510 | + | 188/500 |
| AT2G16760 | - | 216/500 |
| AT2G17030 | - | 337/500 |
| AT2G18969 | - | 138/500 |
| AT2G20740 | - | 429/500 |
| AT2G20825 | + | 53/500 |
| AT2G21780 | - | 374/500 |
| AT2G21910 | + | 477/500 |
| AT2G21950 | + | 25/500 |
| AT2G23160 | - | 391/500 |
| AT2G23310 | - | 264/500 |
| AT2G23350 | + | 255/500 |
| AT2G26230 | + | 104/500 |
| AT2G26740 | + | 398/500 |
| AT2G26830 | + | 474/500 |
| AT2G27940 | + | 242/500 |
| AT2G28370 | + | 430/500 |
| AT2G29290 | - | 275/500 |
| AT2G29380 | + | 233/500 |
| AT2G29580 | + | 27/500 |
| AT2G29700 | - | 217/500 |
| AT2G30230 | - | 97/500 |
| AT2G30670 | - | 224/500 |
| AT2G33480 | + | 258/500 |
| AT2G33585 | - | 320/500 |
| AT2G34340 | - | 143/500 |
| AT2G34630 | + | 33/500 |
| AT2G36895 | + | 405/500 |
| AT2G37250 | + | 113/500 |
| AT2G38130 | + | 329/500 |
| AT2G39780 | - | 44/500 |
| AT2G39970 | + | 152/500 |
| AT2G40420 | + | 85/500 |
| AT2G40540 | + | 374/500 |
| AT2G41770 | - | 78/500 |
| AT2G42670 | - | 309/500 |
| AT2G43070 | + | 384/500 |
| AT2G43080 | - | 342/500 |
| AT2G43420 | - | 110/500 |
| AT2G43540 | - | 82/500 |
| AT2G44620 | - | 485/500 |
| AT2G46390 | - | 46/500 |
| AT2G46505 | - | 143/500 |
| AT2G46540 | + | 11/500 |
| AT2G47490 | + | 429/500 |
| AT2G47970 | + | 14/500 |
| AT3G01850 | - | 97/500 |
| AT3G02340 | - | 489/500 |
| AT3G03070 | - | 345/500 |
| AT3G03120 | + | 314/500 |
| AT3G03720 | + | 472/500 |
| AT3G03860 | + | 225/500 |
| AT3G05010 | - | 394/500 |
| AT3G05970 | + | 365/500 |
| AT3G06190 | + | 147/500 |
| AT3G07100 | + | 361/500 |
| AT3G07568 | - | 192/500 |
| AT3G08650 | - | 435/500 |
| AT3G09085 | + | 31/500 |
| AT3G09180 | + | 298/500 |
| AT3G11200 | - | 239/500 |
| AT3G11330 | + | 446/500 |
| AT3G11660 | + | 394/500 |
| AT3G12070 | + | 235/500 |
| AT3G12100 | - | 370/500 |
| AT3G12760 | - | 217/500 |
| AT3G13520 | + | 162/500 |
| AT3G14075 | + | 178/500 |
| AT3G14595 | + | 340/500 |
| AT3G15640 | + | 180/500 |
| AT3G17611 | + | 430/500 |
| AT3G17770 | + | 9/500 |
| AT3G18430 | - | 488/500 |
| AT3G18520 | + | 17/500 |
| AT3G19980 | + | 192/500 |
| AT3G20740 | + | 371/500 |
| AT3G20890 | + | 219/500 |
| AT3G22530 | + | 337/500 |
| AT3G23605 | + | 341/500 |
| AT3G24610 | + | 168/500 |
| AT3G24730 | + | 104/500 |
| AT3G25220 | - | 391/500 |
| AT3G25585 | + | 192/500 |
| AT3G25620 | + | 62/500 |
| AT3G25800 | + | 91/500 |
| AT3G26680 | + | 79/500 |
| AT3G27890 | - | 107/500 |
| AT3G29200 | + | 415/500 |
| AT3G46000 | - | 139/500 |
| AT3G47990 | - | 88/500 |
| AT3G48140 | + | 291/500 |
| AT3G48150 | + | 248/500 |
| AT3G49390 | + | 189/500 |
| AT3G49590 | + | 120/500 |
| AT3G50810 | - | 320/500 |
| AT3G51300 | + | 364/500 |
| AT3G51430 | - | 166/500 |
| AT3G51460 | - | 483/500 |
| AT3G51990 | + | 104/500 |
| AT3G53780 | - | 433/500 |
| AT3G53970 | - | 160/500 |
| AT3G55050 | + | 480/500 |
| AT3G55390 | - | 472/500 |
| AT3G57785 | - | 383/500 |
| AT3G58730 | - | 84/500 |
| AT3G60020 | - | 235/500 |
| AT3G60720 | + | 52/500 |
| AT3G60980 | + | 142/500 |
| AT3G61070 | - | 227/500 |
| AT3G61680 | + | 340/500 |
| AT3G62260 | - | 454/500 |
| AT3G62830 | - | 16/500 |
| AT3G63260 | + | 467/500 |
| AT4G00585 | + | 275/500 |
| AT4G01410 | + | 340/500 |
| AT4G02860 | + | 377/500 |
| AT4G05018 | + | 130/500 |
| AT4G05050 | - | 482/500 |
| AT4G05590 | - | 138/500 |
| AT4G06676 | - | 101/500 |
| AT4G07390 | - | 95/500 |
| AT4G09030 | + | 344/500 |
| AT4G09580 | + | 247/500 |
| AT4G10330 | + | 188/500 |
| AT4G12230 | + | 192/500 |
| AT4G13870 | - | 144/500 |
| AT4G14010 | + | 263/500 |
| AT4G14160 | - | 94/500 |
| AT4G16444 | - | 237/500 |
| AT4G16450 | + | 337/500 |
| AT4G17530 | - | 382/500 |
| AT4G19040 | - | 433/500 |
| AT4G19640 | - | 190/500 |
| AT4G19645 | - | 398/500 |
| AT4G21810 | + | 465/500 |
| AT4G22240 | + | 258/500 |
| AT4G22550 | - | 452/500 |
| AT4G23530 | + | 300/500 |
| AT4G23710 | - | 209/500 |
| AT4G24690 | - | 133/500 |
| AT4G26310 | - | 360/500 |
| AT4G26320 | + | 98/500 |
| AT4G27320 | + | 116/500 |
| AT4G27780 | + | 377/500 |
| AT4G28060 | + | 342/500 |
| AT4G28730 | + | 365/500 |
| AT4G29580 | - | 281/500 |
| AT4G30010 | - | 488/500 |
| AT4G30510 | + | 423/500 |
| AT4G31300 | - | 109/500 |
| AT4G31615 | + | 422/500 |
| AT4G32130 | - | 68/500 |
| AT4G32870 | + | 243/500 |
| AT4G35850 | - | 253/500 |
| AT4G35860 | + | 229/500 |
| AT4G37830 | - | 181/500 |
| AT4G38250 | + | 139/500 |
| AT4G38260 | + | 485/500 |
| AT4G38630 | - | 190/500 |
| AT5G01800 | + | 234/500 |
| AT5G01850 | + | 205/500 |
| AT5G03290 | - | 466/500 |
| AT5G04260 | + | 138/500 |
| AT5G05930 | + | 305/500 |
| AT5G08060 | + | 100/500 |
| AT5G09370 | - | 227/500 |
| AT5G10400 | + | 121/500 |
| AT5G11090 | + | 75/500 |
| AT5G11280 | - | 93/500 |
| AT5G11950 | + | 101/500 |
| AT5G11960 | - | 123/500 |
| AT5G13430 | + | 459/500 |
| AT5G13440 | + | 479/500 |
| AT5G13580 | - | 133/500 |
| AT5G16370 | - | 449/500 |
| AT5G18310 | + | 342/500 |
| AT5G19050 | - | 259/500 |
| AT5G20100 | - | 37/500 |
| AT5G20120 | + | 487/500 |
| AT5G20650 | - | 342/500 |
| AT5G20740 | - | 207/500 |
| AT5G22360 | + | 269/500 |
| AT5G22730 | - | 269/500 |
| AT5G23490 | - | 265/500 |
| AT5G25050 | + | 397/500 |
| AT5G25770 | + | 433/500 |
| AT5G26720 | + | 298/500 |
| AT5G27710 | - | 116/500 |
| AT5G28050 | + | 94/500 |
| AT5G37920 | - | 287/500 |
| AT5G38650 | + | 374/500 |
| AT5G38670 | - | 215/500 |
| AT5G40810 | + | 284/500 |
| AT5G41130 | - | 143/500 |
| AT5G41800 | + | 103/500 |
| AT5G42890 | - | 148/500 |
| AT5G43280 | - | 219/500 |
| AT5G43780 | + | 107/500 |
| AT5G45350 | - | 489/500 |
| AT5G46380 | + | 352/500 |
| AT5G47200 | - | 456/500 |
| AT5G47860 | + | 156/500 |
| AT5G47880 | + | 440/500 |
| AT5G47910 | + | 299/500 |
| AT5G48020 | + | 404/500 |
| AT5G49610 | + | 3/500 |
| AT5G50850 | + | 387/500 |
| AT5G52570 | + | 429/500 |
| AT5G53030 | - | 179/500 |
| AT5G53330 | + | 353/500 |
| AT5G54250 | + | 93/500 |
| AT5G54900 | - | 259/500 |
| AT5G55530 | + | 359/500 |
| AT5G56180 | - | 299/500 |
| AT5G58240 | + | 70/500 |
| AT5G59430 | + | 143/500 |
| AT5G59890 | + | 450/500 |
| AT5G60580 | - | 477/500 |
| AT5G61900 | + | 375/500 |
| AT5G62200 | - | 485/500 |
| AT5G62575 | - | 107/500 |
| AT5G63220 | - | 348/500 |
| AT5G63620 | - | 317/500 |
| AT5G64250 | - | 268/500 |
| AT5G66760 | + | 98/500 |
| AT5G67010 | + | 172/500 |

**\*** Overlapping sequences are merged to avoid redundancy. As a result some sequences in the final set may be longer than the specified maximum.

Distribution

+

-

Position values represent number of bases from the 5 prime end of the sequence provided to MEME and the length of that sequence. It is these figures expressed as proportions that are used for statistical analysis.

Probability value of comparison of these values to a linear distribution, p = 0.2

The motif is found 185 times on the + stand and 136 times on the - strand.

Probability value that it is evenly distributed between the two strands, p = 0.0036

Move the mouse pointer over the gene ids to highlight the corresponding marker
